# Small GTPase ARL4C Associated with Various Cancers Affects Microtubule Nucleation

**DOI:** 10.3390/biomedicines12122872

**Published:** 2024-12-18

**Authors:** Evgeniia Ulas, Ilya Brodsky, Anton Burakov

**Affiliations:** 1 A.N. Belozersky Institute of Physico-Chemical Biology, Lomonosov Moscow State University, Moscow 119992, Russia; evgeniya.ulas@gmail.com (E.U.); brodskiy-i@yandex.ru (I.B.); 2 Institute of Protein Research of Russian Academy of Sciences, Pushchino 142290, Russia

**Keywords:** ARL4C, ARL7, cancer, RXR/ LXR, microtubules, nucleation

## Abstract

Background/Objectives: The changes in the level of small GTPase ARL4C are associated with the initiation and progression of many different cancers. The content of ARL4C varies greatly between different tissues, and the induction of ARL4C expression leads to changes in cell morphology and proliferation. Although ARL4C can bind alpha-tubulin and affect intracellular transport, the role of ARL4C in the functioning of the tubulin cytoskeleton remained unclear. The aim of the present work is to study this role; Methods: The cells of the following lines were used for the experiments: HeLa (human cervical carcinoma), MCF7 (human breast cancer), U2OS (human osteosarcoma), Vero, BS-C-1, and COS7 (African green monkey kidney). The receptor activation by agonists followed by the preparation of cell lysates, electrophoresis, and immunoblotting, as well as cell fixation and immunofluorescent staining, were used to assess endogenous ARL4C/ABCA1 levels and the microtubule network morphology. The microtubule regrowth technique was performed to estimate the rate of microtubule nucleation, and the overexpression of different ARL4C constructs was used to affect ARL4C activity in the cells; Results: We showed that the changes in the endogenous ARL4C level or the ARL4C activity alter the microtubule nucleation process in the cells; Conclusions: small GTPase ARL4C may serve as one of the regulators of the microtubule nucleation process both in normal and cancer cells.

## 1. Introduction

In recent decades, several studies have linked the changes in the level of small GTPase ARL4C with various cancers and assumed that it may serve as a prognostic marker and a therapeutic target. ARL4C is associated with initiation and progression of leukomyosarcoma [1], glioblastoma [2], lung adenocarcinoma [3,4] and lung squamous cell carcinoma [5], ameloblastoma [6], colorectal cancer [7], liver cancer [8], gastric cancer [9], and renal cell carcinoma [10,11]. However, at the same time ARL4C also acts as a suppressor gene, inhibiting the progression of ovarian cancer [12], and, in addition, the overexpression of ARL4C enhances the sensitivity of the non-small cell lung cancer cells to the erlotinib [13]. The biological role of ARL4C in all these cases remains unclear.

Arl is a subfamily of small GTPases within the Arf family. The Arl4 group (ARL4A, ARL4C, also known as ARL7, and ARL4D) possess a positively charged region in the C-terminal part of the molecule that can determine protein nuclear or plasma membrane localization [14]; moreover, Arl4 GTPases accompany the transport of lipids between the perinuclear space and the plasma membrane. ARL4A, ARL4C, and ARL4D are able to bind the exchange factor Arf nucleotide-binding site opener (ARNO), a GEF for Arf6, and to recruit it to the plasma membrane, thus activating the small GTPase Arf6, that, in its turn, regulates endocytosis, actin dynamics, and cell adhesion [15]. ARL4A has been shown to be involved in the GCC185-dependent recruitment of CLASPs to the Golgi membranes [16]. ARL4D interacts with the microtubule-binding protein EB1, is located at the centrosome, and is involved in the microtubules’ growth on it [17].

Unlike ARL4A, ARL4C (also called ARL7) is not localized at the trans-Golgi network (TGN) [16] and appears to be involved in intracellular cholesterol transport associated with ABCA1-mediated cholesterol secretion [18,19]. It is known that the ARL4C gene is a direct target of the LXR transcription factor in macrophages [20]. ARL4C is considered an important regulator of epithelial multilayered tube-like structure formation in developmental morphogenesis. It has also been determined that the Wnt3a-β-catenin-dependent pathway and EGF-Ras-MAPK pathway cooperatively induce the expression of ARL4C as a direct target gene through the β-catenin, Tcf4, and Ets1 complex, which recruits the histone acetyltransferase and cyclic AMP-responsive element binding protein (CREB)-binding protein (CBP) to enhance histone H3 acetylation and activate ARL4C transcription. During the process of epithelial tube-like structure formation, cell proliferation must be promoted. Increased ARL4C expression via EGF and Wnt3a growth factors stimulation induces the nuclear localization of the Yes-associated protein (YAP) and the transcriptional co-activator with a PDZ-binding motif (TAZ) [21], which are transcriptional coactivators in the Hippo signaling pathway that promotes cell proliferation [22]. Another important condition for epithelial tubulogenesis is to stimulate cellular migration. The increased expression of ARL4C after EGF and tWnt3a treatment also leads to small GTPase Rac activation [21], apparently through ARNO binding and recruitment to the plasma membrane and subsequent Arf6 activation [15]. Rac small G-proteins are known to activate WAVE, which binds actin monomers, recruits Arp2/3 complexes, and, thus, induces lamellipodia formation through actin filaments branching and rapid actin polymerization at the leading edge of the cell during membrane protrusion for mesenchymal types of cell movement [23,24]. The ARL4C-mediated activation of Rac1 also inhibits Rho activity [21], which suppresses an amoeboid type of movement by decreasing actomyosin contractility at stress fibers [24] via the inhibition of myosin 2 light chain phosphorylation [25]. Also, ARL4C itself binds and affects filamin-A, an actin-binding protein, that provides cross-linking between actin filaments into a three-dimensional network by promoting activation of Cdc42, and thereby regulates filopodium formation during cell migration [26]. Actin cytoskeletal rearrangement is required for cell morphological changes that promote epithelial cell migration, proliferation, and differentiation towards surrounding mesenchymal tissue to form epithelial tubular structures [27,28]. These tubular structures’ morphogenesis provides functional units for many epithelial organs, including the intestine, kidneys, salivary glands, lungs, mammary glands, pancreas, and liver [29]. In particular, in the developing kidney epithelial ureteric buds enter the surrounding metanephric mesenchyme, branch rapidly, and form kidney collecting ducts [30]; however, this process is inhibited by the knockdown of ARL4C [21].

The variety of ARL4C possible roles in the cells correlates with the fact that the content of both ARL4C mRNA and protein itself varies greatly between different tissues. Thus, human ARL4C expresses at high levels in the brain and at lower levels in the spleen, kidney, thymus, lymph nodes, esophagus, uterus, stomach, and intestine of adult people [31], while mouse ARL4C expresses in a relatively high level in the brain, stomach, submandibular glands, kidney, lungs, intestine, tooth buds, and hair follicles at 13th–15th-day embryos [21]. It seems that one or more functions of ARL4C acquire additional importance during cancer progression, which is reflected in the changes in its level in many cancer cells. It has been shown previously that the level of ARL4C in cultured human cervical carcinoma (HeLa) cells could be increased through the activation of the liver X receptor (LXR) and retinoid X-receptor (RXR) or cholesterol loading [18].

The cellular function of ARL4C related to microtubule cytoskeleton has not been studied yet: it has only been shown that ARL4C could bind α-tubulin independently of the GTP- or GDP-binding state and affects intracellular transport of transferrin from the early to the recycling endosomes [32]. In the present work, we investigated the biological role of ARL4C and found that changes in its level or activity affect the nucleation of microtubules and thus it could serve as a regulator of the tubulin cytoskeleton.

## 2. Materials and Methods

### 2.1. Cell Cultures and RXR/LXR Activation

Human cervical carcinoma (HeLa) and MCF7 (human breast cancer) cells were kindly provided by Dr. S.E. Dmitriev, U2OS (human osteosarcoma) cells were kindly provided by Dr. I.M. Terenin, cultured green monkey kidney Vero, BS-C-1, and COS7 cells were taken from the lab stocks. The cells were cultured in DMEM/F12 (1:1) culture medium (PanEco, Moscow, Russia) supplemented with 2 mM L-glutamine (PanEco, Moscow, Russia), 10% FBS (PanEco, Moscow, Russia), and 50 μg/mL gentamicin at 37 °C with 5% CO_2_. Cell lysate preparation and immunofluorescent staining were carried out, for this the cells were grown for 24 h before the experiment after seeding on Petri dishes or coverslips, correspondingly. To affect LXR/RXR, the cells were treated with T0901317 at 0.25 to 10 μM (Sigma–Aldrich, Saint Louis, MO, USA) and bexarotene from 0.25 to 2.5 μM (Sigma–Aldrich, Saint Louis, MO, USA) (LXR and RXR agonists respectively). The T0901317- and bexarotene-containing culture media were replaced with fresh media every 24 h.

### 2.2. Preparation of Lysates, Electrophoresis, and Immunoblotting

Cell extracts were prepared with the addition of lysis buffer (200 mM Tris-HCl pH = 6.8, 4% SDS, 25% glycerol, 1 M β-mercaptoethanol, and a complete protease inhibitor cocktail (Roche Applied Science, Basel, Switzerland)). The proteins were separated in 12% (when working with ARL4C) or 6% (when working with ABCA1) SDS-polyacrylamide gel electrophoresis and were transferred to a nitrocellulose membrane (GE Healthcare Life Solutions, Boston, MA, USA). Then, the membrane was blocked with 5% skimmed milk in TBST for 1 h. Rabbit polyclonal antibody against ARL4C (Novus Biologicals, Centennial, CO, USA), mouse monoclonal antibody against GAPDH (ThermoFisher Scientific, Waltham, MA, USA), rabbit polyclonal antibody against ABCA1 (Novus Biologicals, Centennial, CO, USA), mouse monoclonal antibody against α-tubulin, and clone DM1A (Santa Cruz Biotechnology, Dallas, TX, USA) were used as primary antibodies. Horseradish peroxidase-labeled anti-rabbit IgG (KPL, Gaithersburg, MD, USA), horseradish peroxidase-labeled anti-mouse IgG antibody (Jackson ImmunoResearch Laboratories, Bar-harbor, ME, USA), phosphatase-labeled goat anti-rabbit IgG (KPL, Gaithersburg, MD, USA), and phosphatase-labeled goat anti-mouse IgG (KPL, Gaithersburg, MD, USA) were used as secondary antibodies. Peroxidase and phosphatase were developed using a WesternBright ECL HRP substrate (Advansta, San Jose, CA, USA) and BCIP/NBT Phosphatase Substrate (KPL, Gaithersburg, MD, USA), correspondingly.

### 2.3. Microtubules Regrowth Experiments, Cell Fixation, and Immunofluorescent Staining

For microtubule disassembly, the cells were treated with 3 μg/mL of nocodazole (2 h at 37 °C, further 1 h at 0 °C, and finally 30 min at 37 °C). Then, nocodazole was washed out with a warm cell culture medium, and the cells were incubated at 37 °C for 45 s prior to fixation. Alternatively, nocodazole was washed at room temperature for 2 or 5 min. After microtubule recovery, cells were treated with the buffer containing 50 mM imidazole, 50 mM KCl, 0.5 mM MgCl_2_, 1 mM EGTA, 0.1 mM EDTA, 30% glycerol, and 0.5% Triton X-100 for 1 min to extract soluble tubulin. The cells were fixed with 100% methanol (5 min at −20 °C) and 3% paraformaldehyde (20 min at +4 °C). For immunostaining, primary antibodies were used at 1–5 μg/mL (1:200 dilution of stock solution). Secondary antibodies were used at 5 μg/mL. The incubation time with both primary and secondary antibodies was 1 h at room temperature in the dark in a wet chamber. Mouse monoclonal antibodies against α-tubulin, clone DM1A (Santa Cruz Biotechnology, Dallas, TX, USA), and Alexa Fluor™ 488-conjugated anti-mouse-IgG secondary antibodies (Jackson ImmunoResearch Laboratories, Bar-harbor, ME, USA) were used. The coverslips were mounted in Aqua PolyMount (Polysciences, PA, USA).

For imaging, a Zeiss LSM900 confocal microscope was used (provided by the Moscow State University Development Program). The obtained data were processed using ZEN 3.5 blue edition software (Version 3.5.093.00002) (Zeiss, Oberkochen, Germany).

### 2.4. DNA Constructs

To obtain ARL4C cDNA, total RNA was isolated from Vero cells using an RNeasy kit (74104, Qiagen, Hilden, Germany). First-strand cDNA was synthesized with Revert Aid reverse transcriptase (EP0441, ThermoFisher Scientific, Waltham, MA, USA) and random hexanucleotide primers. Arl4C cDNAs were amplified with specific primers with the terminal sites for restriction endonucleases (forward—GACGGTACCGGCAACATCTCCTCTAACATC, reverse—ATAGGATCCTTACCGCTTCTTCTTCTG) and was further cloned into the pTagRFP-C vector (Evrogen, Moscow, Russia). cDNAs were verified using automated DNA sequencing. Site-directed mutagenesis was performed using Change-IT Site-Directed Mutagenesis kit (78480, Affymetrics, Santa Clara, CA, USA). To generate the GTP-locked, constitutively active mutant, ARL4C[Q72L], a specific primer (forward—GGACGTGGGCGGCCTGGAGAAGCTGCGGCC) was used. To generate the GDP-locked dominant-negative mutant, ARL4C[T27N], a specific primer (forward—ACTCGGCCGGCAAGAACACGGTGCTCTACC) was used. All the DNA constructs were verified by automated DNA sequencing. High Fidelity PCR Enzyme Mix (K0191), thermosensitive calf intestinal alkaline phosphatase, and T4-ligase were from ThermoFisher Scientific (Waltham, MA, USA). Restriction endonucleases were from ThermoFisher Scientific (USA) or SibEnzyme (Novosibirsk, Russia). Primers were from either Syntol (Moscow, Russia) or Evrogen (Moscow, Russia). The Plasmid Miniprep kit, PCR purification, and gel purification kit were from Evrogen (BC021, BC041S, Moscow, Russia) or Qiagen (12125, Hilden, Germany). CAMSAP3-GFP plasmid was the same as in [33].

## 3. Results

### 3.1. Elevation of Endogenous ARL4C Level in HeLa Cells Is Accompanied by Enhanced Microtubules Nucleation in the Cytoplasm

Unlike ARL4A and ARL4D, the link of ARL4C with the tubulin cytoskeleton has never been shown previously; however, since it binds α-tubulin and affects intracellular transport we decided to check it. There are many inhibitors and modulators of different upstream members of signaling pathways regulating the expression of ARL4C, such as the inhibitors and activators of the PI3K/Akt/mTOR signaling pathway, MAPK signaling pathway, and Wnt/β-catenin signaling pathway (see in the Discussion section); however, unfortunately, there are no available compounds targeting ARL4C directly. We reproduced the effect of the stimulation of ARL4C protein synthesis using the agonists of the LXR/RXR transcription factor in HeLa cells and then studied how that affects the microtubules.

**Figure 1:**
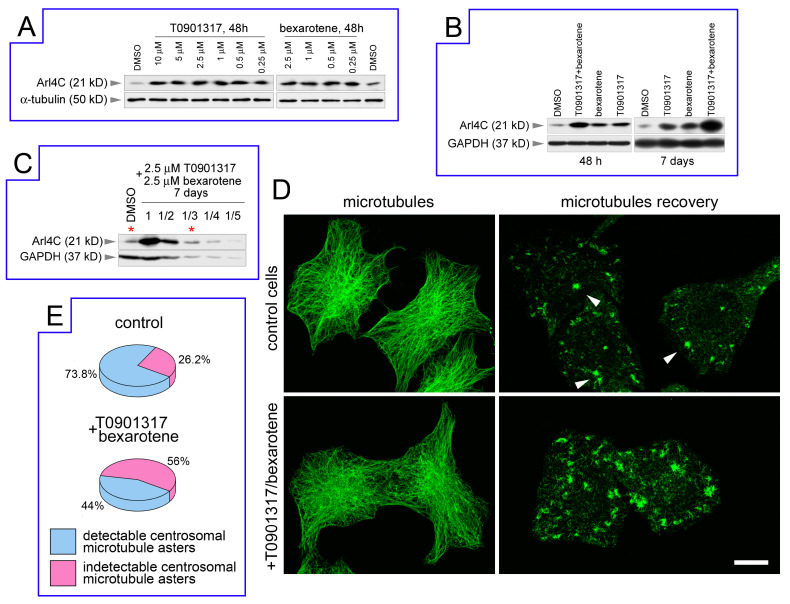
Increased level of endogenous ARL4C results in activation of cytoplasmic nucleation of microtubules in HeLa cells: (**A**) Increase in ARL4C level in HeLa cells treated with T0901317 or bexarotene in a dose-independent manner. (**B**) Treatment of HeLa cells with the combination of T0901317 and bexarotene (2.5 μM each) and prolonged exposure result in an increased level of ARL4C. (**C**) Treatment of HeLa cells with the combination of LXR/RXR ligands results in a three-fold increase in ARL4C level. Asterisks mark lines with comparable amounts of ARL4C in control lysate and in the lysate of treated HeLa cells diluted 1:3. (**D**) (left): Microtubules in control HeLa cells and in cells treated with T0901317/bexarotene (2.5 μM) for 48 h. (right): 45 s of microtubule recovery in control HeLa cells and in cells treated with T0901317/bexarotene (2.5 μM) for 48 h. Arrows point to centrosomal microtubule asters. Scale bar is 10 μm. (**E**) Statistical analysis of microtubule regrowth shown in (**D**); *n* = 99 for control cells and 125 for T0901317/bexarotene-treated cells in the two independent experiments.

First, we used the LXR agonist T0901317 from the original work of Engel and co-authors [18]. Since the combination of RXR and LXR ligands results in a more prominent activation of the LXR/RXR transcription factor [34], we added the RXR activator, bexarotene. The HeLa cell culture was incubated for 48 h with various concentrations of T0901317 or bexarotene added to the culture medium. The concentration of T0901317 ranged from 0.25 μM to 10 μM; the bexarotene concentration ranged from 0.25 μM to 2.5 μM. All used concentrations did not have a significant effect on cell growth and vitality. After the incubation period, cell lysates were prepared. According to the results of immunoblotting, the level of ARL4C protein in HeLa cells treated with T0901317 or bexarotene significantly increased (Figure 1A). Interestingly, the increase did not depend on the concentration of T0901317 and bexarotene in the range used. We observed a more pronounced effect on ARL4C level in HeLa cells with the combination of T0901317 and bexarotene (2.5 μM each) compared to their separate usage (Figure 1B). In addition, when the exposure time was increased from 48 h to 7 days while maintaining the selected concentrations of agonists (2.5 μM), an even more pronounced increase in the level of ARL4C was observed (Figure 1B). This approach resulted in an increase in Arl4C level by approximately three times compared to the baseline (Figure 1C).

Next, we studied the effect of ARL4C level elevation on microtubules. We stained the cells with antibodies to α-tubulin and found that, in the control and treated HeLa cells, the morphology of the tubulin cytoskeleton was similar: the microtubules network was more or less radial and microtubules were gathered in the central region without the clearly defined point of convergence marked the centrosome (Figure 1D). It is well known that such microtubule-organizing centers as the centrosome and the Golgi are usually located close to each other in the central region of the cell and they are difficult to be separated spatially. To study the microtubule nucleation in detail, we performed depolymerization and recovery experiments, which had been previously proven to be informative in this regard [35,36]. Cells that were fixed after depolymerization without the microtubule recovery demonstrated the complete disassembly of the tubulin cytoskeleton, and the staining with antibodies to α-tubulin revealed the centrioles only. After the nocodazole washout, the microtubules rapidly grew in the cell. Along with the centrosomal asters of the microtubules, we observed numerous short single cytoplasmic microtubules, as well as additional small asters of cytoplasmic microtubules extending from non-centrosomal nucleation sites. We explored several recovery times and chose 45 s for these experiments when numerous microtubules were already appearing in the cytoplasm; however, they had not yet merged into a dense network. As a result, we found a clear phenotypic difference between control cells and cells treated with T0901317/bexarotene: the number of cytoplasmic asters noticeably increased after the treatment, and the centrosomal asters could not be easily distinguished among all of them (Figure 1D,E). Indeed, in the control cells, the centrosomal microtubule asters were clearly detected among smaller cytoplasmic nucleation clusters in 73 out of 99 cells, while, in cells treated with T0901317/bexarotene, centrosomal asters were distinguishable only in 55 out of 125 cells in the two independent experiments (Figure 1E).

Thus, we found that an increase in the endogenous ARL4C level in HeLa cells caused by the activation of the LXR/RXR receptors was followed by the emergence of multiple microtubule nucleation sites in the cytoplasm.

### 3.2. The RXR/LXR-Dependent, ARL4C-Involving Pathway May Be Inactive in the Cells

**Figure 2:**
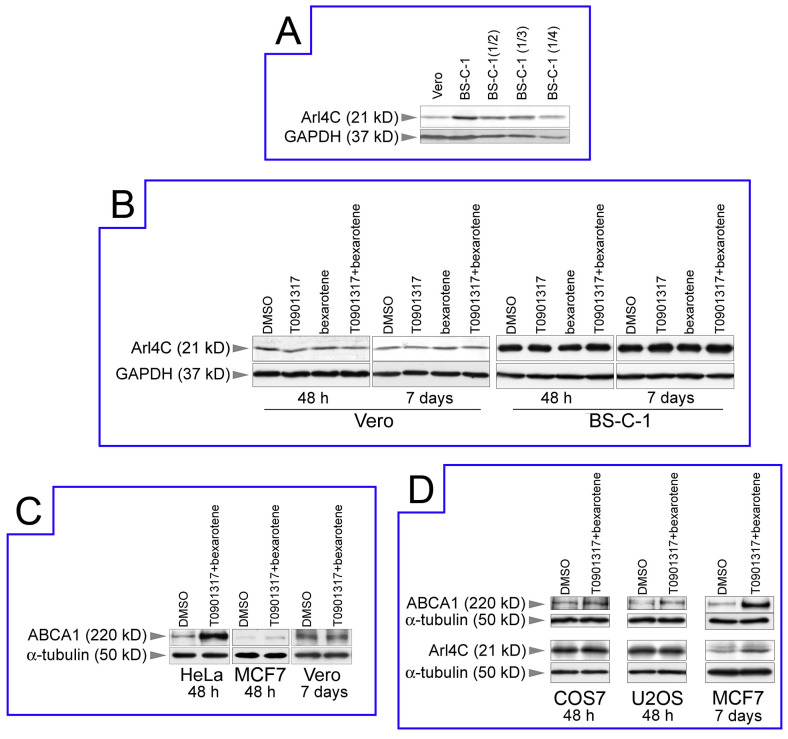
Treatment using LXR/RXR ligands does not lead to an increase in the endogenous ABCA1 or ARL4C level in many cell lines: (**A**) Titration of BS-C-1 lysate demonstrates that the ratio of levels of ARL4C in Vero and BS-C-1 cells is 1:4.5. (**B**) The level of ARL4C is not increased in both Vero and BS-C-1 cells even after 7 days of treatment with T0901317 and bexarotene. (**C**) LXR/RXR-dependent pathway is activated in HeLa cells and weakly activated in MCF7 cells after 48h exposure to 2.5 µM T0901317/bexarotene; however, not in Vero cells even after prolonged treatment. (**D**) Neither ABCA1 nor ARL4C levels increase after treatment of COS7 and U2OS cells with RXR/LXR ligands. Long-term treatment of the MCF7 cells results in only a slight increase in the ARL4C level.

The observed alterations in microtubule nucleation following changes in endogenous ARL4C level may indicate the redistribution of microtubule nucleation activity between the centrosomal and cytoplasmic microtubule-organizing centers. It is well known that the relative contribution of the different MTOCs varies greatly depending on cell type [37]. We found earlier that, in two cultured African green monkey cell lines, Vero and BS-C-1, the balance between centrosomal and non-centrosomal microtubule nucleation differs, and the level of mRNA encoding ARL4C also differs by approximately 4.6-fold; herewith, the higher ARL4C mRNA level in BS-C-1 cells is correlated with greater non-centrosomal microtubule nucleation [33]. We then decided to check whether a high level of ARL4C protein and non-centrosomal microtubule nucleation are indeed related to each other, i.e., whether Vero and BS-C-1 cells differed in ARL4C protein levels in accordance to differences in mRNA levels. Titration of cell lysates following Western blotting demonstrated that, indeed, the ratio of ARL4C protein in Vero and BS-C-1 cells matched the ratio of the corresponding mRNAs and is approximately 1:4.5 (Figure 2A). Thus, the level of endogenous ARL4C in various cultured green monkey cells, as well as in experiments with human HeLa cells, correlates with the non-centrosomal microtubule nucleation level.

The next stage of our study was to elevate the level of endogenous ARL4C through the activation of the RXR/LXR-dependent pathway in various cells and investigate the following changes in the microtubules’ nucleation. We treated Vero cells, where the role of non-centrosomal MTOC is relatively low, with T0901317 and bexarotene to increase the level of ARL4C as we did with HeLa cells. Surprisingly, it turned out that neither 2.5 µM T0901317 nor 2.5 μM bexarotene, or their combination, had any effect on the ARL4C level in Vero cells, neither after 48 h nor even after 7 days of exposure (Figure 2B). Similarly, there was no effect on the ARL4C level after the same treatment of BS-C-1 cells (Figure 2B). It should be noted that in the cells of both these lines, the basic level of ARL4C was much higher than in HeLa cells. Based on the transcriptome analysis, we can approve that mRNAs encoding the LXR/RXR receptors are present in both these cells (NCBI Sequence Read Archive project id PRJNA862476). Therefore, we decided to test the level of ABCA1 protein in the cells treated with T0901317/bexarotene, because the ABCA1 gene is the target of the transcription factor LXR/RXR [38].

We found that the LXR/RXR-dependent pathway was indeed activated in HeLa cells, and a noticeable increase in ABCA1 was observed after the experimental treatment (Figure 2C). However, in another human cancer cell line (breast cancer cell line MCF-7), this effect was very weak, and this pathway was not activated at all in Vero cells even after 7 days of exposure to LXR/RXR ligands (Figure 2C). We checked the possibility of activating this pathway and changing the level of endogenous ARL4C in another widely used African green monkey kidney cells (COS7) and human cancer U2OS osteosarcoma cell line. We found that, in these cells, this signaling pathway is inactive (Figure 2D). The long-term exposure of MCF7 cells by 2.5 μM T0901317/bexarotene for 7 days ultimately led to an increase in the level of ABCA1 and some increase in Arl4C (Figure 2D), but did not affect the distribution of microtubule-nucleation activity. From all this, we can conclude that exposure by RXR/LXR ligands is not a universal way to change the level of endogenous ABCA1 and ARL4C. The fact that the bright effect of LXR/RXR transcription factor agonists on the levels of these two proteins was observed only in HeLa cells leads us to the conclusion that this mechanism may be cell-specific rather than a general cellular mechanism. Therefore, to study the role of ARL4C in the process of microtubule nucleation in other cells, we had to resort to the usage of genetic constructs.

### 3.3. ARL4C Inhibition Violates the Microtubule Nucleation at the Centrosomes

For the next experiments on activation and inhibition of ARL4C, we chose Vero cells, where the microtubules are organized mainly by the centrosomes, and the possible alterations in the non-centrosomal microtubule nucleation due to changing of the ARL4C activity would be clearly visible. The level of endogenous ARL4C protein in these cells is high enough, based on our previous experiments with cell lysates; therefore, we first studied its distribution in Vero cells. We immunostained the cells with antibodies against ARL4C and α-tubulin to visualize both the protein of interest and microtubules.

**Figure 3:**
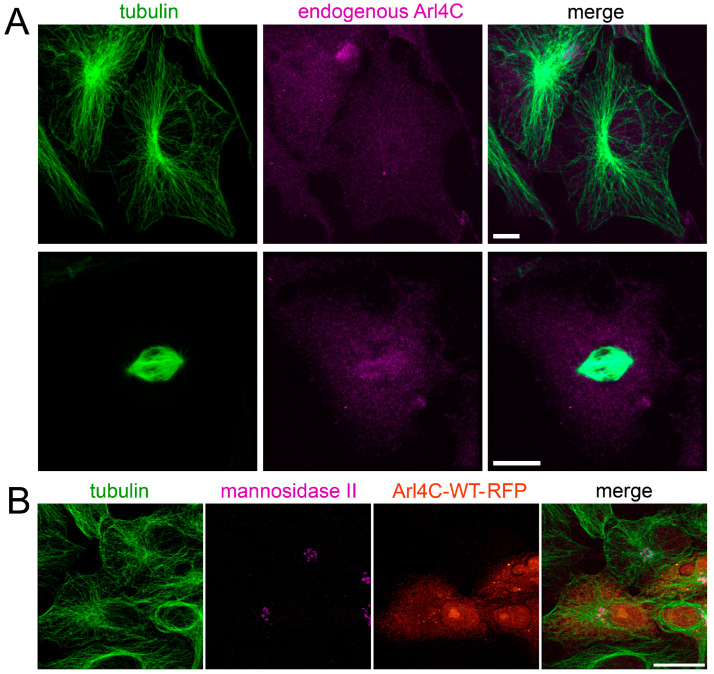
Both endogenous and exogenous ARL4C do not have a specific localization pattern in the cytoplasm: (**A**) Vero cells immunostained with antibodies to tubulin DM1A (green) and to ARL4C (magenta). Scale bar is 10 µm (**B**) Morphology of microtubules in cells expressing ARL4C-WT-RFP (red). Immunostaining of transfected Vero cells with antibodies to tubulin DM1A (green) and to the Golgi marker mannosidase II (magenta). Scale bar is 20 µm.

We found that in interphase cells, endogenous ARL4C does not co-localize either along the microtubules or with anything resembling a centrosome or Golgi ribbon at the area of their convergence (Figure 3A). In mitotic cells, we could also definitively state that endogenous ARL4C does not co-localize with the centrosomes. An analysis of the individual focal planes of stained mitotic cells did not allow us to state with certainty that ARL4C co-localizes with spindle microtubules, although staining for ARL4C was usually higher in the spindle region (Figure 3A bottom). After that, we transfected the cells with a plasmid encoding wild-type ARL4C and examined both the ARL4C-WT distribution and the morphology of microtubules in transfected cells. We found that the microtubule system did not differ significantly from that in control cells and stayed generally radial, with microtubules diverging from the center (Figure 3B). The central region of the Vero cells usually contains both the centrosome and Golgi ribbon [33], and, just as in the case of the endogenous ARL4C, we saw no evidence of overexpressed ARL4C-WT co-localization with microtubules or Golgi.

**Figure 4:**
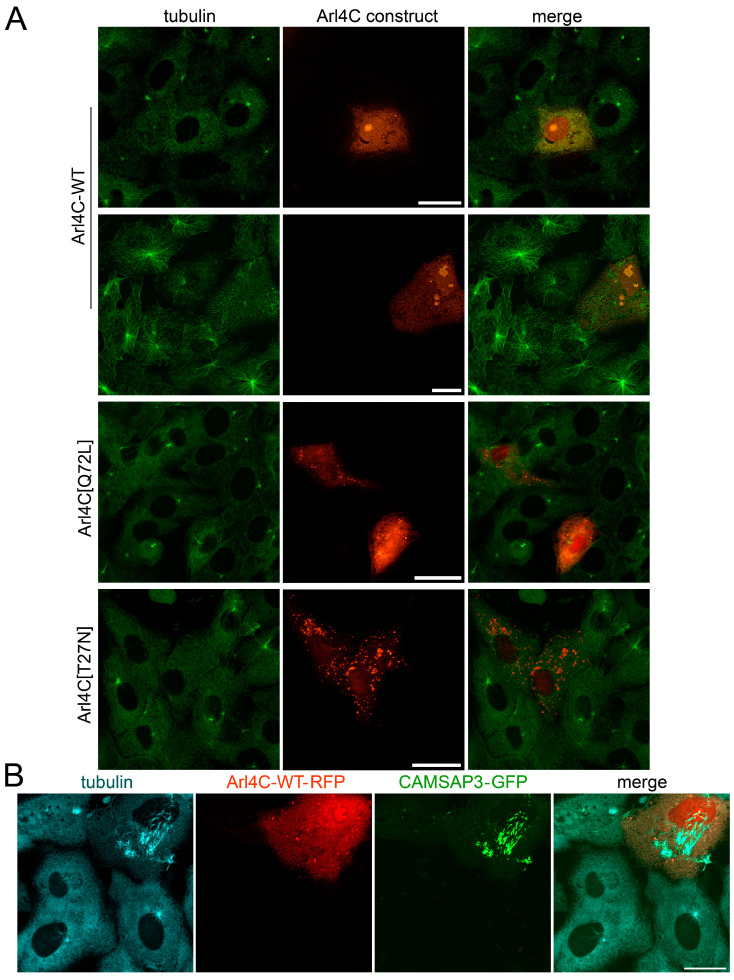
ARL4C inhibition disrupts the microtubule nucleation at the centrosomes in Vero cells: (**A**) Microtubule regrowth in Vero cells expressing ARL4C-WT-RFP, ARL4C[Q72L]-RFP and ARL4C[T27N]-RFP (red). Immunostaining with antibodies to tubulin DM1A (green). Scale bars represent 20 µm. (**B**) Microtubule regrowth in Vero cells co-expressing ARL4C-WT-RFP (red) and CAMSAP3-GFP (green). Immunostaining with antibodies to tubulin DM1A (blue). Scale bar represents 20 µm.

In order to study the role of ARL4C activity in microtubule nucleation in detail, we conducted microtubule regrowth experiments in the cells expressing the wild-type ARL4C-WT, a GDP-locked, dominant-negative mutant ARL4C[T27N] and a GTP-locked constitutively active mutant ARL4C[Q72L]. We found that the expression of ARL4C-WT or constitutively active ARL4C[Q72L] had no visible effect: the centrosomes remained mostly the only microtubule nucleation sites both in control and transfected cells at the early stages of microtubule regrowth and later (Figure 4A). Sometimes the cells expressing ARL4C-WT demonstrated some reduction in the size of centrosomal microtubule asters. However, the expression of dominant-negative ARL4C[T27N] led to a sharp decrease in the rate of nucleation at the centrosomes (Figure 4A). This indicates that although the overexpression of wild-type or constitutively active ARL4C cannot increase the non-centrosomal microtubule nucleation in Vero cells where the centrosomes play a major role, ARL4C still is involved in the regulation of nucleation in these cells.

To exclude the possibility that overexpression of used genetic constructs has cytotoxicity that could non-specifically negatively affect the microtubule nucleation process throughout the cell, we stimulated the non-centrosomal nucleation of microtubules in ARL4C-expressing Vero cells using overexpression of CAMSAP3, which is important for the non-centrosomal organization of microtubules [39]. We have already seen before that the overexpression of CAMSAP3 in Vero cells led to the appearance of abundant stable cytoplasmic bundles of microtubules [33]. Now we co-expressed CAMSAP3 and ARL4C-WT and performed microtubule regrowth experiments. We found that the presence of exogenous ARL4C does not interfere with the cytoplasmic nucleation of microtubules caused by CAMSAP3-GFP expression (Figure 4B). Therefore, the effect on centrosome activity in Vero cells observed in our experiments is specific. Thus, the small GTPase ARL4C could affect the microtubule nucleation both in cells where its basic level is low and could be regulated by the RXR/LXR signaling pathway and in cells with an initially high level of this protein and centrosome-oriented microtubule organization process.

## 4. Discussion

In this work, we studied the biological role of small GTPase ARL4C associated with several types of cancer. We showed for the first time that the elevation of the endogenous ARL4C level through the RXR/LXR-dependent pathway in human cervical carcinoma HeLa cells is accompanied by an increased non-centrosomal nucleation of microtubules. At the same time, ARL4C inhibition through the expression of corresponding genetic construct in green monkey kidney cells leads to the weak nucleation of microtubules at the centrosomes. These data suggest that ARL4C may serve as one of the controllers of the microtubule nucleation process both in normal and cancer cells.

By now, a number of data have already been obtained about ARL4C level regulation in cancer cells. Particularly, the expression of ARL4C in such cancer cell lines as HCT116 colorectal cancer cells, A549 lung cancer cells, primary hepatocellular carcinoma HLE cells, and S2-CP8 cells (sequential passage of a human pancreatic cancer cell line through the lung of a nude mouse) is upregulated by the Wnt3a/β-catenin-dependent pathway and/or EGF- Ras-MAPK pathway (reviewed in [40]). Loss of phosphatase and tensin homolog deleted on chromosome 10 (PTEN) in primary human glioblastoma (GBM) stabilized ARL4C due to the AKT/mTOR pathway-mediated inhibition of ARL4C ubiquitination. Targeting PTEN potently inhibited GBM tumor progression in vitro and in vivo, whereas overexpression of ARL4C reversed the tumor progression impaired by PTEN overexpression [2]. ARL4C expression is downregulated via the inhibition of the AKT pathway in lung adenocarcinoma A549 and 95-D cells, whereas exposure to benzo(a)pyrene (a carcinogen in smoke) increased ARL4C expression in human airway epithelial 16HBE cells via AKT activation. In addition, the chemotherapy drug hydroxycamptothecin (HCPT) could decrease ARL4C expression levels by inhibiting the activation of the AKT pathway in A549 and 95-D cells [4]. Ursolic acid (UA) treatment significantly decreased the ARL4C protein level in human colon cancer and inhibited the AKT/mTOR signaling pathway. Overexpression of ARL4C reversed the inhibitory effect of UA on the invasion and migration of HCT-116 and SW480 cells. Also, UA and an AKT signaling pathway inhibitor (LY294002) induced the ubiquitination of the ARL4C protein, which was reversed by a proteasome inhibitor MG-132 [41]. In the present study, we used the effect of the stimulation of ARL4C protein synthesis by the agonists of the LXR/RXR transcription factor first demonstrated by Engel and co-authors [18] and found that this method is not universal among the different cell lines.

The observed differences in the regulation of ARL4C activity and its impact on the microtubule nucleation process in our study could be explained by the differences in the relative contribution of various MTOCs in different cells. The role of the centrosome as a microtubule-organizing center varies greatly depending on cell type. Thus, in RPE1 cells (retinal pigment epithelium), approximately half of all the microtubules are nucleated not at the centrosome but at the Golgi membranes [42]. The non-centrosomal organization determines the specific architecture of the microtubule system and in general all the polarized intracellular transport in neurons. It was shown that the non-centrosomal microtubules can fulfill some specific functions; for example, the Golgi-derived microtubules serve as fast cargo tracks required for cell migration [43]. The ability to redistribute the microtubule-organizing activity among the cytoplasm when necessary could be useful for the cell’s response to various external events; however, the ways to achieve this remain mostly unclear. Perhaps ARL4C contributes to the regulation of this process. It is obvious that the presence of such regulation is not necessary for every cell, and this is precisely what can explain the tissue-specific expression of ARL4C.

In contrast to the regulation of the actin cytoskeleton, the molecular mechanisms of ARL4C functioning as a regulator of the microtubule nucleation are still completely unclear. The possibility that these signaling pathways intersect in some way cannot be ruled out, given that microtubule-organizing centers may also serve as centers for organizing the actin microfilaments [44]. An intriguing question is what causes the increased level of ARL4C observed in various cancer cells. Arf family proteins tend to retract their interswitch region in the GDP-bound state so that the N-terminal amphipathic helix packs into a pocket formed by the retracted interswitch region, and conformational change upon GTP binding leads to the interswitch region ejecting the N-terminal amphipathic helix from the pocket, which provides the protein’s membrane association [45]. The interswitch region of ARL4 proteins is longer than that of other Arf family proteins, which means that it may not be able to form a retractile conformation in the GDP-bound state [46]. Considering the fact that ARL proteins have both GTP-binding activity and nucleotide exchange rate considerably higher than that of the other Arfs [31], it can be concluded, that the biological activity of ARL4 proteins may be regulated by its expression levels rather than switches between GDP- and GTP-bound states [47]. Specific guanine-nucleotide exchange factors (GEFs) and GTPase-activating proteins (GAPs) that regulate the GDP- or GTP-binding states of ARL proteins have not yet been identified [40]. At the same time, it remains completely unclear whether the change in ARL4C level/activity is a consequence of disruptions in the centrosomes’ work, which are often observed in cancer cells due to the violation of the centriolar cycle, or some kind of compensatory mechanism that corrects these disruptions.

## 5. Conclusions

Our research showed that small GTPase ARL4C associated with various cancers may serve as one of the regulators of the microtubule nucleation process both in normal and cancer cells.

## Data Availability

The original contributions presented in this study are included in the article. Further inquiries can be directed to the corresponding author.
